# Supplementary material for: Modulation of Cellular Response to Different Parameters of the Rotating Magnetic Field (RMF)—An In Vitro Wound Healing Study
Source: Int J Mol Sci. 2021 May 28;22(11):5785. doi: 10.3390/ijms22115785 (PMC8199476; doi:10.3390/ijms22115785)
Supplement: Supplementary file 1 [file ijms-22-05785-s001.zip › ijms-1182253-supplementary.pdf]

Supplementary Materials

Modulation of Cellular Response to Different Parameters of the Rotating Magnetic Field (RMF)—An in Vitro Wound Healing Study

Magdalena Jedrzejczak-Silicka[1], Marian Kordas [2] , Maciej Konopacki [2] and Rafał Rakoczy [2,*]

[1]Laboratory of Cytogenetics, West Pomeranian University of Technology in Szczecin, Klemensa Janickiego 29, 71 - 270 Szczecin, Poland; mjedrzejczak@zut.edu.pl

[2]Faculty of Chemical Technology and Engineering, West Pomeranian University of Technology in Szczecin, Piastow Avenue 42, 71 - 065 Szczecin, Poland; mkordas@zut.edu.pl; mkonopacki@zut.edu.pl

*Correspondence: rrakoczy@zut.edu.pl

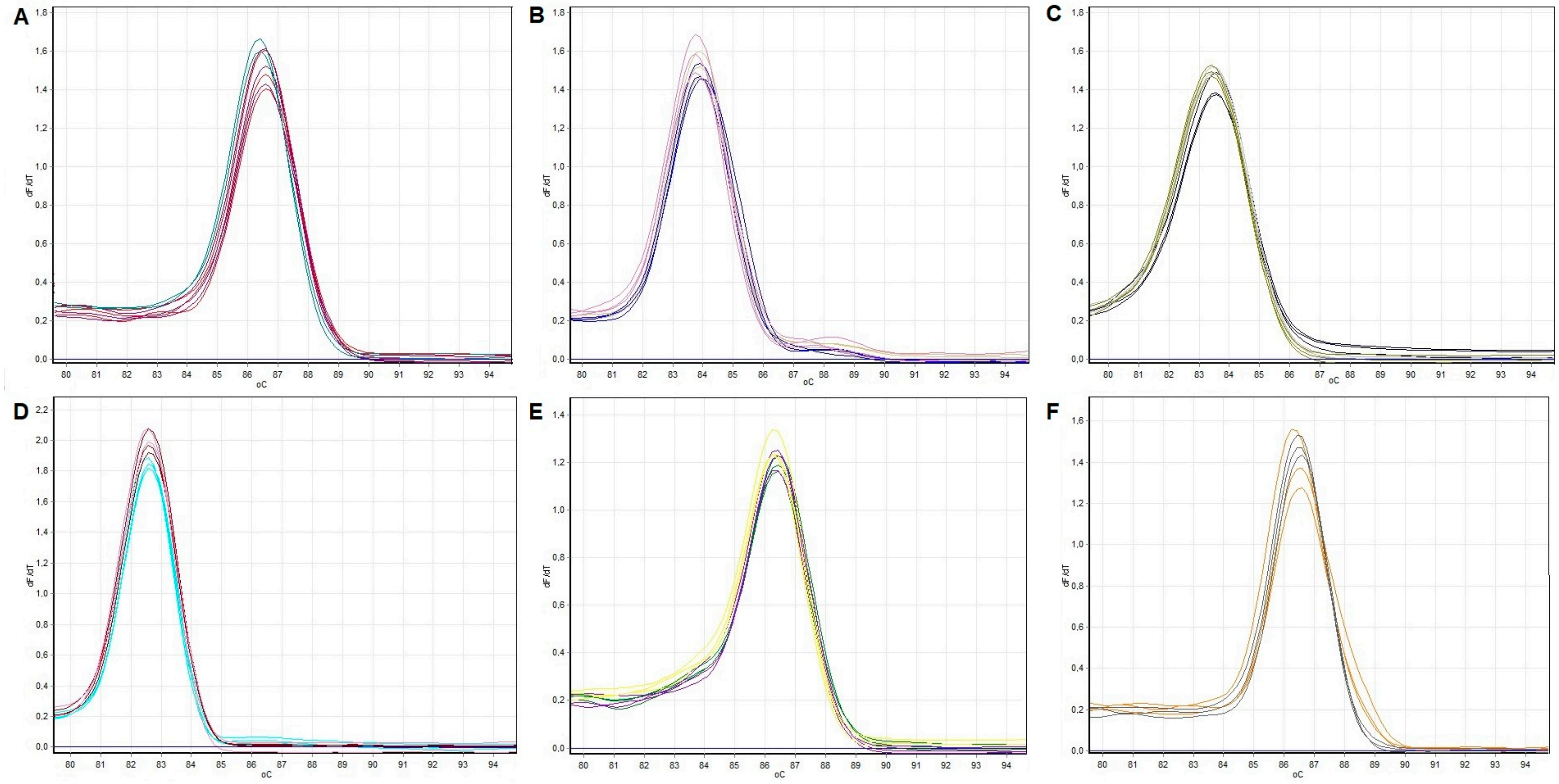

Figure S1. Melt curve analysis for *col1a1* (A), *col3a1* (B), *cdc42* (C), *Rac1* (D), *actb* (E) and *gapdh* (F) in samples of L929 cells.

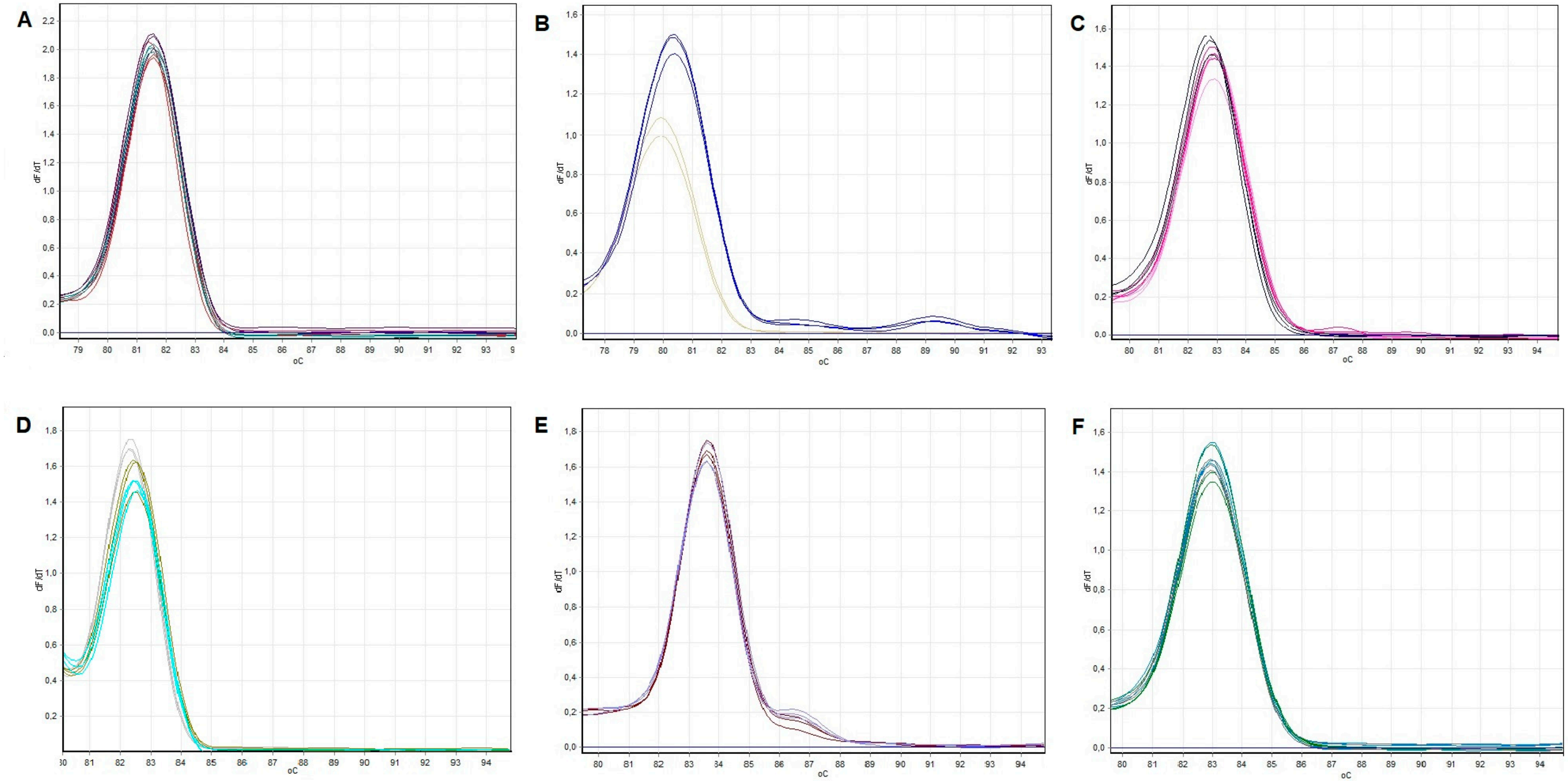

Figure S2. Melt curve analysis for *KRT10* (A), *KRT14* (B), *CDC42* (C), *RAC1* (D), *ACTB* (E) and *GAPDH* (F) in samples of HaCaT cells.
